# Streamlining emergency nursing care post-pandemic: A lean approach for reducing wait times and improving patient and staff satisfaction in the hospital

**DOI:** 10.1186/s12912-025-02759-w

**Published:** 2025-04-22

**Authors:** Azza Hassan Mohamed Hussein, Ebtsam Aly Omer Abou Hashish, Basmaa Ahmed Abd-Elghaffar, Nancy Sabry Hassan Elliethey

**Affiliations:** 1 Nursing Administration & Healthcare Management, Nursing Administration Department, Faculty of Nursing, Alexandria University, Alexandria, Egypt; 2 College of Nursing, King Saud bin Abdul-Aziz University for Health Sciences, Jeddah, Saudi Arabia; 3 King Abdullah International Medical Research Center, Jeddah, Saudi Arabia

**Keywords:** Lean methodology, Emergency department, Waiting time, Service time, Length of stay, Patients, Healthcare providers

## Abstract

**Background:**

In emergency departments (EDs), long wait times and overcrowding are major challenges, worsened by the pandemic's increased patient volumes and demands. Lean methodology could offer a structured approach to reduce inefficiencies, improve care quality, and support nursing staff. Aim of the study: This study aims to evaluate the impact of applying a Lean approach to optimize emergency nursing care post-pandemic within an ER setting.

**Methods:**

This study utilized a mixed-methods design in the ER of a private hospital in Egypt. Data collection involved three Lean tools: the voice of the process observation sheet, which tracked the journeys of 100 patients; voice of customer structured interviews, conducted with 90 patients to assess satisfaction with waiting times; and voice of business interviews, held with 64 staff members to evaluate satisfaction with available resources. Additionally, a cause-and-effect analysis was conducted and summarized in an A3 report, identifying key factors contributing to extended wait times.

**Results:**

The average wait time in the emergency department was 157.87 min, making up 77.7% of the total length of stay. The consultation phase accounted for the longest delays, with an average wait of 92.46 min. Patient satisfaction with waiting times was moderate (61.74%), while staff satisfaction with resources was higher (71.09%), but only 53.1% were satisfied with patient wait times. Key causes of delays included non-compliance with triage protocols (95.0%), lack of care pathways (90.3%), and insufficient bed capacity (83.1%). An A3 report proposed strategies to reduce wait times and enhance satisfaction.

**Conclusion:**

This study highlights waiting times as a major challenge in EDs, significantly impacting service quality, patient outcomes, and nursing staff workload. Lean-based strategies, such as standardized triage and improved care pathways, are essential to reducing delays and enhancing both patient care and staff satisfaction in the post-pandemic healthcare environment.

**Supplementary Information:**

The online version contains supplementary material available at 10.1186/s12912-025-02759-w.

## Introduction

The post-pandemic era has not served as a "strategic inflection point" for transformative change in many healthcare systems. Instead, some nations have reverted to pre-pandemic practices, seemingly overlooking the crisis's enduring impact [1]. Emergency departments (EDs), in particular, face significant challenges in navigating the aftermath of COVID-19 while continuing to operate in an increasingly unstable and complex healthcare environment [2, 3]. The growing demands on EDs, including overcrowding, treatment delays, diminished quality and safety of care, and inefficient resource utilization, have further strained patient flow and care delivery processes [4, 5].

These challenges, compounded by rising patient volumes and healthcare complexities, exacerbate issues such as prolonged wait times, inadequate resources, and overcrowding, ultimately compromising patient outcomes and care quality [6, 7]. To address these challenges, hospitals have increasingly adopted management frameworks such as Lean Management (LM) and supply chain practices to drive sustainable performance improvements. These approaches enable hospitals to adapt rapidly to the evolving healthcare landscape by emphasizing continuous quality enhancement, operational flexibility, and timely responsiveness to patient needs [8–10].

This study aimed to evaluate patient flow within an emergency department (ED) by employing a Lean-integrated approach to measure key performance indicators (KPIs) across three dimensions: the voice of the process (wait time, service time, and length of stay), the voice of the customer (patient satisfaction), and the voice of the business (staff satisfaction with resources). Additionally, the study proposed an A3 report outlining targeted strategies to reduce waiting times and optimize ED operations. The findings offer actionable insights into modifiable operational factors, equipping hospital leaders with evidence-based strategies to streamline patient flow, address inefficiencies, and improve overall ED service quality.

### Literature review

Lean methodology focuses on identifying and eliminating waste, defined as non-value-added processes, to streamline patient flow in emergency departments (EDs) and enhance value for patients [11]. In healthcare, value is determined by activities that improve care quality and patient outcomes while meeting needs within a defined cost and timeframe. Removing waste from healthcare processes creates value, with waste encompassing any activity that does not benefit patients or causes delays in their treatment [12–14]. In the ED, examples of waste include waiting times for care or the next treatment phase, which disrupt patient flow. Eliminating such inefficiencies promotes smoother workflows, improves care quality, and enhances safety and efficiency [15].

Lean methodology has been adopted to tackle persistent ED challenges like delays, overcrowding, and medical incidents [14, 16]. It has demonstrated potential in improving customer-focused outcomes such as faster response times, streamlined processes, and better patient satisfaction. However, further empirical validation is required to fully substantiate these benefits [16, 17]. Prioritizing patient needs, a core principle of Lean, is fundamental to delivering effective care and securing resources from donors and funding agencies [18].

### Conceptual framework

This research is guided by Lean methodology principles aimed at optimizing ED operations. Spear and Bowen [19] outlined four core principles for operational improvement: standardizing work to reduce ambiguity, fostering connections among team members, establishing seamless workflows, and empowering staff to resolve issues using a scientific approach [19, 20]. Central to this framework is eliminating waste, with process mapping as a vital tool for analyzing non-value-added activities and improving performance [21]. Widely applied in EDs, process mapping assesses patient and logistics flows to identify inefficiencies [22, 23]. Substantial evidence supports the application of Lean Management (LM) in EDs, showing improvements in patient flow, reduced waiting times, minimized waste, and a focus on continuous improvement as a driver of organizational change [22–26].

#### Lean approach and patient flow

Enhancing patient flow in the emergency department (ED) is achieved by adopting Lean strategies that integrate the Voice of Customer (VOC), Voice of Process (VOP), and Voice of Business (VOB) perspectives. The VOP reflects the process's performance capability in meeting patient expectations and needs, while the VOC captures patient requirements comprehensively. It defines key inputs for setting design specifications, establishes a common language for team collaboration, and supports innovation in healthcare services [18]. Analyzing VOP helps healthcare providers identify necessary process improvements to align better with patient expectations, ultimately streamlining care and enhancing satisfaction [17].

The VOB, representing staff input, focuses on formal and informal expressions of ideas, suggestions, and alternative approaches to improve organizational processes. This input addresses inefficiencies and enhances functionality, fostering a more effective and responsive ED environment [27]. By integrating VOP, VOC, and VOB, healthcare organizations adopt a holistic approach to improving patient flow, service efficiency, and overall quality in the ED.

#### Length of stay, waiting time, and service time

Emergency departments (EDs) often face delays in patient care due to extended waiting times, which frequently surpass the actual time spent receiving healthcare services, such as interventions or interactions with healthcare professionals [28]. Service time represents the total duration of active care, beginning when a service is initiated and concluding upon its completion [28, 29].

To address these delays, several countries, including England, Australia, Scotland, Stockholm, and Northern Ireland, have established waiting time standards to reduce the overall length of ED visits. For example, England's National Health Service (NHS) mandates that 95% of ED patients must be seen, treated, and either admitted or discharged within four hours [30, 31].

Implementing Lean methodology with regular monitoring of length of stay, waiting time, and service time is crucial, particularly in the post-COVID-19 context. This approach facilitates more efficient ED operations, ensuring timely care delivery and improving the overall patient experience [11].

#### Significance of the study

The ED operates within a complex network of processes, where upstream and downstream activities significantly impact patient flow throughout the hospital system [32]. Effective triage is crucial for identifying and prioritizing patients requiring urgent care, positioning the ED as a critical frontline in both routine healthcare and crisis response. Studying key metrics such as waiting time, service time, and length of stay (LOS) provides actionable insights for optimizing resources and improving care quality [29]. While prior research has explored individual variables, such as LOS during the COVID-19 pandemic [32, 33] or waiting times [34–36], few studies have analyzed these metrics collectively. This study addresses this gap by examining the full patient journey from registration to disposition, offering a comprehensive evaluation of ED performance post-COVID-19. Using Lean methodology, it identifies inefficiencies and provides targeted strategies to enhance patient flow and care quality [22, 29].

For nursing, the findings are particularly significant. Nurses, as key figures in ED operations, manage triage, patient care, and coordination. By examining delays in waiting time, service time, and LOS, this study equips nurses with insights to address inefficiencies, improve patient satisfaction, and ensure safety. Applying Lean principles allows for streamlining workflows, reducing waste, and fostering a more patient-centered environment [13, 14]. Moreover, the research empowers nurses to drive system-level improvements, positioning them as leaders in care quality and operational efficiency. By mitigating delays and promoting continuous improvement, the study supports nurses in reducing burnout, enhancing job satisfaction, and delivering evidence-based care that improves outcomes and teamwork [9, 18].

### Aim of the study

This study aims to evaluate the impact of applying a Lean approach to optimize emergency nursing care post-pandemic by assessing key performance indicators, including wait times, patient satisfaction, and staff satisfaction, within an emergency department setting.

### Research questions

1. What are the sources of wasted waiting time in ED processes?
2. What is the Voice of Customer (VOC) concerning patient satisfaction with waiting times for services received in the ED?
3. What is the Voice of Business (VOB) regarding waiting times, their causes, and potential improvements?
4. What insights can be gathered from the Voice of Process (VOP) regarding the current patient flow journey in the ED?
5. What recommendations can be made in an A3 problem-solving report to enhance waiting times in the ED?

## Methods

### Study design

This study employed a mixed-methods approach, combining quantitative and qualitative data to evaluate Lean-inspired improvement initiatives in the ED. This design integrates the strengths of both approaches, offering a comprehensive analysis to enhance the understanding of ED performance and processes [37].

### Study setting

The study was conducted in the ED of a leading private hospital in Egypt, renowned for its advanced medical care and state-of-the-art laboratory services. Serving both business-to-business (B2B) and business-to-customer (B2C) needs, the hospital provides a wide range of medical and surgical specialties. The ED, chosen as the focal point for this study, is equipped with 10 beds and staffed by a dedicated team of healthcare providers committed to delivering efficient and timely emergency care in the Egyptian healthcare context.

### Study participants

Participants for this study were selected using convenience sampling, comprising 100 patients who visited the ED during morning and afternoon shifts. Eligibility criteria required participants to be 18 years or older, capable of understanding the study protocol, and able to respond to study questions. Non-urgent cases, such as those seeking routine exams, investigations, or medication refills, were excluded to maintain the study's focus on emergency care scenarios.

The sample size was calculated using G*Power software (version 3.1.9.7), a widely recognized tool for power analysis and sample size determination. The calculation was based on a power of 80% (β = 0.20), a confidence level of 95% (α = 0.05), and an expected medium effect size (0.5). This effect size is typically recommended for operational studies aiming to detect moderate improvements in metrics such as wait times and patient satisfaction.

To capture the Voice of Business (VOB), the study included a convenience sample of 64 ED healthcare providers with a minimum of 6 months of ED experience. This group comprised 26 nurses, 14 physicians, 12 nursing and medical managers/leaders, 2 quality coordinators, and 10 clerical workers, all of whom participated voluntarily.

### Study measurement tools (instruments)

Three data collection tools, developed by the researchers based on a review of current literature [22, 23, 38–40], were utilized in this study:

#### Tool 1: voice of customer (VOC)

This tool assessed patients' satisfaction with their waiting time in the ED. It consists of two parts:

- *General Information Sheet:* This section gathers demographic and other relevant information, such as date of admission, sex, educational qualification, occupation, residence, payer type, arrival method to the ED, and medical/surgical history.
- *Patient Satisfaction:* This section includes 11 items to evaluate satisfaction levels among 90 patients regarding waiting times across various ED processes, including intervals from arrival to registration, registration to first assessment, first assessment to consultation, consultation to intervention, and intervention to disposition decision. Responses were recorded on a 5-point Likert scale, ranging from strongly dissatisfied (1) to strongly satisfied (5), with an overall score range of 11 to 55. Scores of 42–55 (≥ 66.66%) indicate a high level of satisfaction, 27–41 (≥ 33.33% to < 66.66%) a moderate level, and 11–26 (< 33.33%) a low level of satisfaction [22].

#### Tool 2: voice of business (VOB)

This tool was used to assess the satisfaction levels and perspectives of ED staff (nurses, physicians, quality coordinators, clerical workers) and management (nursing and medical leaders) on ED services and processes, as well as their recommendations for reducing waiting time. It consists of four parts:

- *General Information Sheet for VOB:* This section gathers demographic and professional information about ED staff, such as age, employment position, years of experience, and tenure in the current organization.
- *VOB Satisfaction Structured Interview:* Comprising 12 items to assess staff and managers’ satisfaction with ED resources, structure, equipment, supplies, waiting time, communication, and staffing/beds. Responses were scored on a 3-point Likert scale from strongly dissatisfied (1) to strongly satisfied (3), with a total score range of 12 to 36. Scores of 28–36 (≥ 66.66%) reflect high satisfaction, 21–28 (≥ 33.33% to < 66.66%) moderate satisfaction, and 12–20 (< 33.33%) low satisfaction. [23]
- *VOB Perspectives on ED Improvement:* A semi-structured interview with staff was conducted to identify causes of ED waiting times and propose solutions. Staff were asked two questions: one about the causes of waiting time delays, categorized into manpower, environment, management, methods/process, and machinery (illustrated through a cause-and-effect diagram), and another about recommendations for reducing waiting times.

#### Tool 3: voice of process (VOP)

**Fig. 1:**
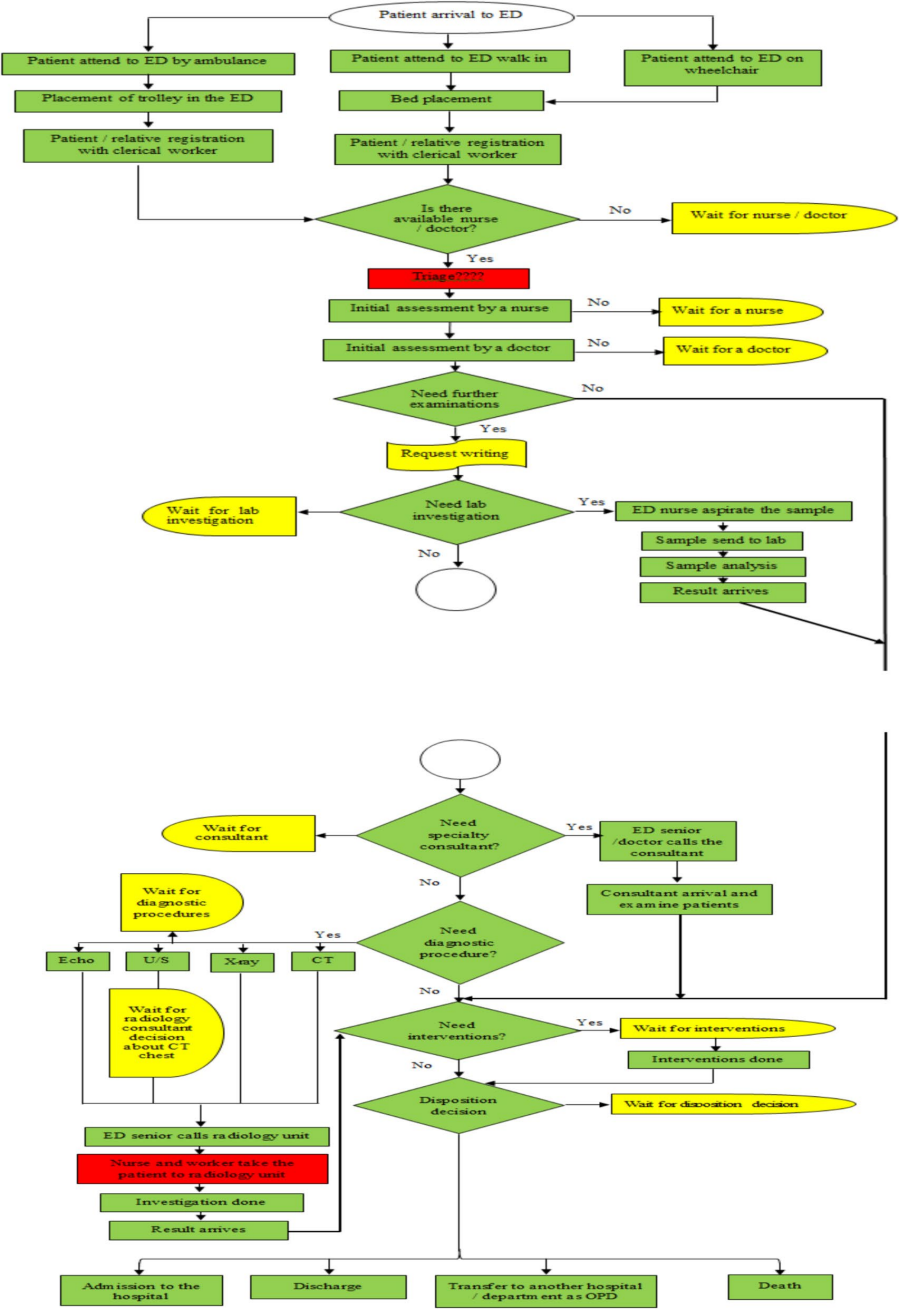
The actual process map for current patient flow in the ED

**Fig. 2:**
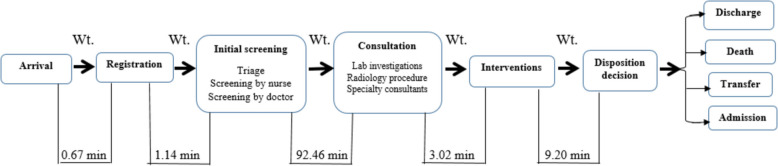
Waiting time within the current patient flow in the ED

**Fig. 3:**
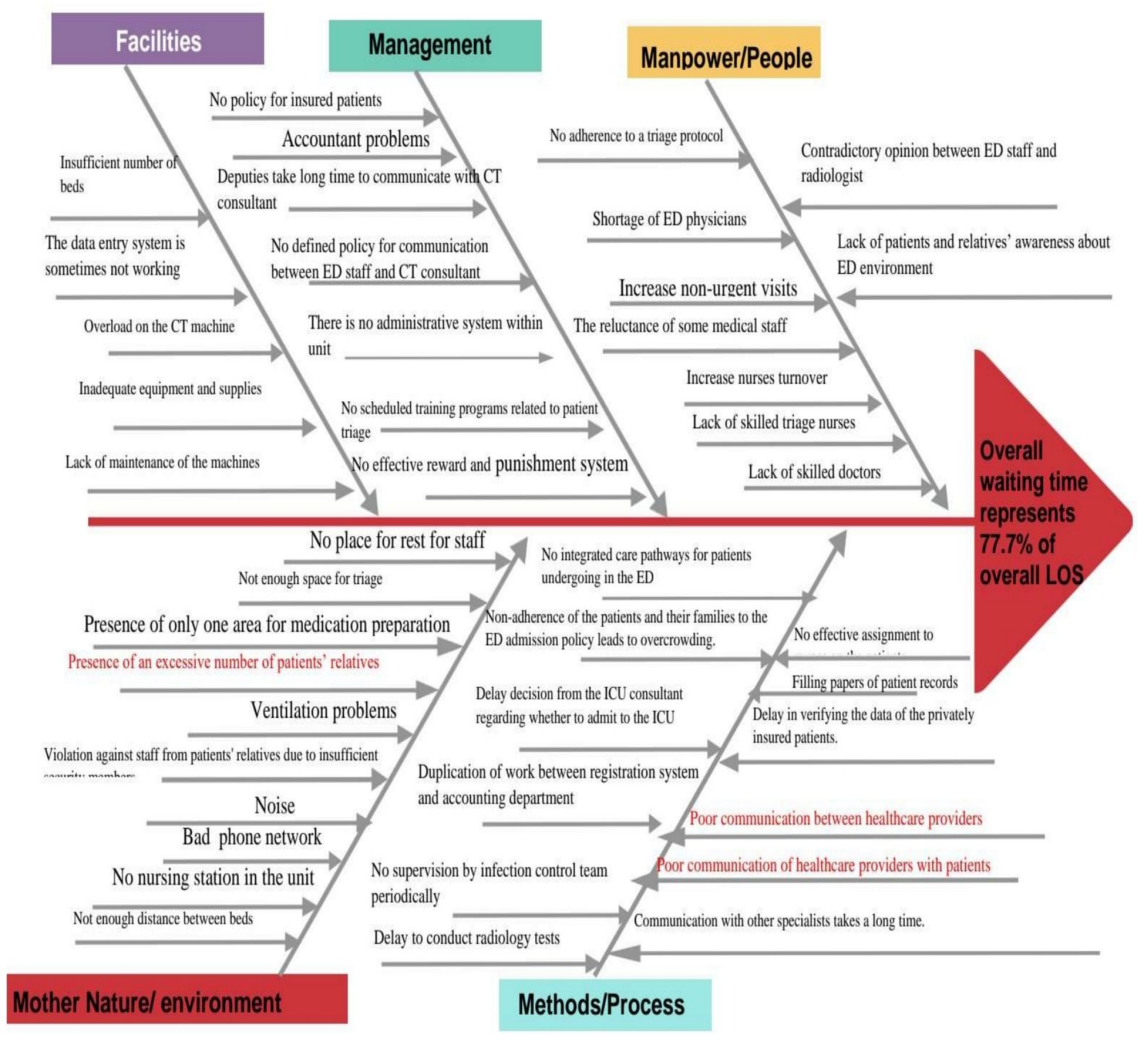
The proposed A3 proplem solving report for enhancing waiting time in ED

An observation sheet was used to trace patients’ journeys in the ED, recording each process’s duration, including waiting, service, and length of stay (LOS) (see Figs. 1 and 2). This data was used to create a process map and an A3 report proposal. Based on literature, the researchers developed the A3 Problem-Solving Sheet (Proposal A3 Report), which describes the current process condition, waiting times at each stage, cause analysis, and an implementation plan proposal. The A3 report was formatted on one side of an A3-sized (11 × 17 inch) sheet of paper, flowing top-to-bottom on the left side and then top-to-bottom on the right (see Fig. 3). It was organized into eight sections: Title, background, current condition process map, goal statement, root cause analysis, plan details/countermeasure, implementation, and follow-up. The first five sections are on the left side of the A3 sheet, with the remaining three on the right side. Once completed, the A3 report included sponsor acceptance lines for sign-off.

### Validity and reliability

To ensure cultural and linguistic compatibility with Egyptian patients and ED staff, all tools were initially translated into Arabic, accommodating varied educational levels. A panel of five academic experts then rigorously evaluated the translated tools for content validity and linguistic clarity, assessing each item individually for relevancy, comprehensiveness, and comprehension. Minor adjustments were made to improve clarity, followed by a back-translation into English by linguists to enhance accuracy and reduce potential validity threats. The original development, validity, and reliability of the tools were previously discussed by Al Owad [39] and Al Owad et al. [22, 23] in their foundational work. However, their discussion focused on the conceptual and practical application of these tools without reporting specific statistical values for validity or reliability. In our study, we addressed this gap by conducting a detailed content validity assessment and internal consistency reliability analysis. The tools demonstrated strong validity, with content validity indices of 0.83, 0.84, and 0.703 for VOC, VOB, and VOP, respectively. Internal consistency reliability was confirmed through Cronbach's alpha coefficients of 0.92 for VOC, 0.88 for VOB, and 0.85 for VOP, with all values significant at p ≤ 0.05. In addition, a pilot study was conducted with a sample of 10 patients and 6 healthcare providers, representing 10% of the study population. The findings indicated that no modifications were necessary, as the tools were user-friendly and easily understood by both groups.

### Data collection process and phases

The data collection process was divided into four main phases:

### First phase

#### Engagement with ED management and staff

Researchers held individual meetings with medical and nursing directors to explain the study's purpose, gaining their cooperation. An initial tour of the ED with the nurse manager helped familiarize researchers with the ED environment and processes. ED staff were introduced to the study, Lean concepts, and the significance of the research for ED improvement.

#### Preparation and initial observation

Before beginning actual observations, researchers engaged in friendly conversations with participants, assuring them of confidentiality and that no information would be disclosed that could cause harm. This phase aimed to build a positive relationship with the medical team. Patients were selected based on inclusion criteria, ensuring attention was not drawn to cases under observation. Observers did not have dedicated observation times; instead, they conducted observations as opportunities arose, sometimes facing delays due to factors such as staff shortages..

### Second phase (Voice of Process—VOP)

#### Process flow chart and gemba walk

The first step involved creating a process flow chart using the Lean Gemba Walk tool to illustrate the patient pathway in the ED. The chart documented clusters of processes, responsible personnel, required resources, and time spent on waiting, service, and length of stay (LOS). A Gemba walk blank sheet captured firsthand observations, helping to document insights for process improvement (see Figs. 1 and 2).

#### Preliminary observation

In August 2023, a preliminary assessment was conducted, shadowing the journey of 10 patients using a record note, pencil, and stopwatch to document the workflow and time sequence of each emergency procedure.

#### Detailed observation of patient flow

Researchers observed the ED patient journey through five main processes: arrival, registration, initial assessment (nurse and physician), consultation (investigations and screening), interventions based on diagnosis, and disposition decision. Observations were recorded from 07:00 to 22:00, and clinical data were gathered from patient records. Observations of 100 patients spanned 872 sub-processes from September 2023 to March 2024, leading to the creation of the actual process map (see Figure 1). While the Australian Triage Scale (ATS) was adopted in the ED, it was not consistently followed for the observed patients.

#### Patient satisfaction interview (VOC)

Researchers conducted structured interviews using the VOC tool with 90 patients or their authorized family members to gauge satisfaction with waiting times encountered throughout their ED journey. The interview was conducted at the disposition decision stage after patients received the necessary treatment.

### Third phase (Voice of Business—VOB)

#### Staff structured interview

Researchers conducted structured interviews with ED staff at their work site using the VOB tool to assess satisfaction with ED resources, such as bed capacity, equipment, unit design, and waiting times.

#### Cause analysis and root cause identification

**Fig. 4:**
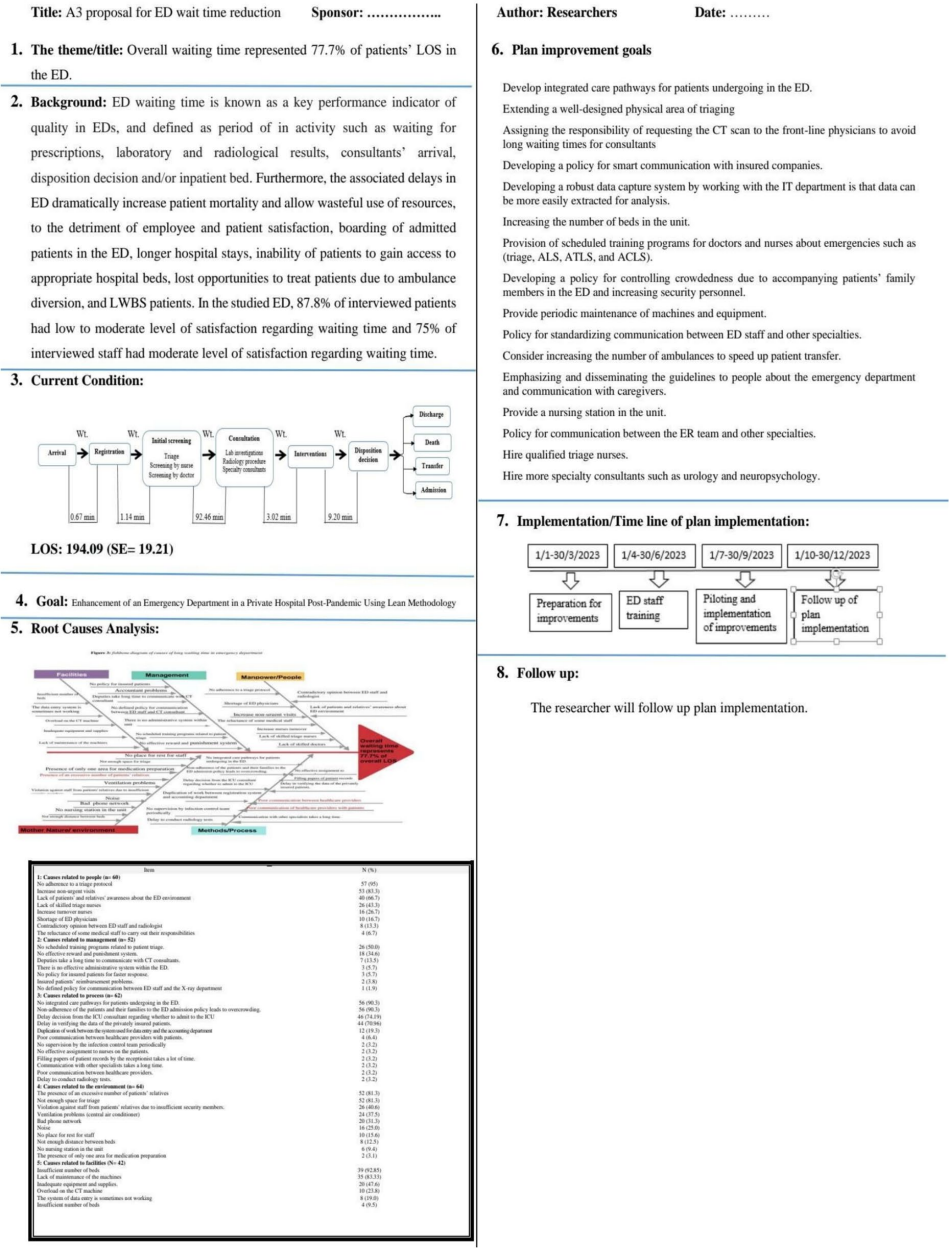
Fishbone diagram of causes of long waiting time in emergency department

Based on literature and observations, researchers identified five major causes of waiting time: people, management, environment, processes, and ED facilities (Fig. 4). A fishbone diagram was developed to capture these root causes. Through a semi-structured interview, staff shared their perspectives on these causes using the VOB tool.

#### Multi-voting methodology for improvement suggestions

Researchers divided participants into four groups, each with eight members. Flip charts were used to list reported causes and suggested improvements. Participants then voted on their top five improvements. Each participant awarded five points to their top choice, four to their second, and so on. After discussion, a final vote confirmed the prioritized suggestions.

### Fourth phase (A3 Proposal Development)

#### Identifying key issues and root causes

Through direct observation and data from A3 sheets completed by ED staff, researchers identified inefficiencies such as unclear patient pathways, redundant registration processes, excessive paperwork, and overcrowding by family members. Other issues included a lack of distinction between urgent and non-urgent conditions, a shortage of physicians and nurses, and inadequate documentation.

#### Proposal A3 report

The A3 report addressed issues such as policy, management practices, organizational processes, and other areas needing careful consideration. It began with a thematic title describing the proposal's focus on reducing the waiting time, which accounted for 77.7% of patient LOS in the ED. The "Background" section highlighted the impact of waiting time on patient satisfaction and staff perspectives.

#### Current condition and goal statement

The "Current Condition" section included a process flow diagram, detailing the patient journey from arrival through disposition. The "Goal" section provided a qualitative target for improving patient flow.

#### Root cause analysis

Using Ishikawa (fishbone) diagrams, 5 Whys analysis, and Pareto charts (see Figure 4), researchers examined the root causes based on data from semi-structured staff interviews.

#### Countermeasures and implementation plan

Researchers developed a plan with detailed countermeasures to address each identified root cause, listing recommendations to meet the improvement goal (Table 5 & Fig. 4).

### Data analysis

Upon completion of data collection, the data were analyzed to assess the components of the ED reception processes, with a primary focus on identifying waste in terms of waiting time across current patient flow processes. Data were entered and analyzed using IBM SPSS software version 26. The Kolmogorov–Smirnov test was applied to check for the normality of data distribution. The results showed non-significant p-values for both patient satisfaction (0.065) and Voice of Business (VOB) satisfaction (0.754), indicating that the data followed a normal distribution (see supplementary files). For quantitative data, descriptive statistics included the range (minimum and maximum), mean, and standard deviation. A significance level of p ≤ 0.05 was utilized for all analyses. Qualitative data were summarized using frequencies and percentages.

### Ethics approval and consent to participate

This study received ethical approval from the Ethical Research Committee (ERC) of the Faculty of Nursing, Alexandria University (IRB00013622). In addition, permission was obtained from the hospital administration before data collection commenced. Participants were provided with detailed information about the study's objectives, emphasizing the confidentiality of their data and the assurance of anonymity and privacy. Written informed consent was obtained from each participant before their involvement in the study. To maintain confidentiality, all questionnaires were assigned unique code numbers, and data were used exclusively for research purposes. Participants were explicitly informed of their right to withdraw from the study at any time without providing a reason. To facilitate observation in the study setting, consent for the researcher's presence was also obtained from the first-line nurse manager. Patient privacy was prioritized throughout the research process, with stringent measures in place to safeguard the confidentiality of the data collected. The autonomy of participants was respected, and their decision to participate or not was fully acknowledged and honored.

## Results

### Demographic and professional characteristics

A total of 100 patients were observed in the ED of the studied hospital. Based on hospital registration data, 89% of patients arrived on foot, 9% by ambulance, and 3% by wheelchair. Although no formal triage system was in place, the researcher conducted triage assessments for study participants. Of all visits, 50% of patients were classified into the less urgent triage levels III, IV, and V, while the remaining 50% were categorized into more urgent levels I and II. Triage distribution showed that 48% of cases were imminently life-threatening, 25% potentially life-threatening, 13% less urgent, 12% potentially serious, and 2% immediately life-threatening. Reasons for ED visits included neurology and cardiology issues (each at 32%), sepsis and suspected appendicitis (21%), respiratory issues (8%), and accidents (7%) (Supplementary Table ).

Of the 100 patients, 90 were interviewed for their satisfaction. The mean age was 52.69 years (SD = 17.73) and the majority of patients were male (68%) and from urban areas (86.66%), with 13.33% from rural regions. In terms of educational level, 31.11% completed secondary school, 22.22% held a university degree or higher, 20% had a high school education, 16.6% had a primary education, and 1% were uneducated. Occupations were distributed as follows: 27.7% were housewives, 27.7% retired, 21.11% public employees (professionals), 21.11% private sector workers, and 2.22% students. Most patients (83.33%) were self-paying, while 16.66% had private health insurance (Supplementary Table 2).

### Patient journey and process flow in ED

Figures 1 illustrates the patient journey through the ED, consisting of five main phases from admission to disposition. The first phase, Admission and Registration, begins when the patient enters the ED and continues through the registration process, marking the start of their formal engagement with the system. Following this, the Initial Assessment phase involves three sub-processes: a preliminary assessment by a nurse, an evaluation by a doctor, and, if necessary, triage, helping to determine the patient’s priority level and immediate care needs. The third phase, Consultation, begins with either a referral for consultation or the initiation of a diagnostic procedure, further assessing the patient’s condition. The Intervention phase follows, where necessary medical interventions are carried out, beginning with the intervention order and concluding upon completion of all required procedures. Finally, the Decision Disposition phase involves a disposition decision based on the patient’s condition and reimbursement guidelines, with options including transfer to another hospital, discharge, admission, or, in rare cases, death.

### Analysis of length of stay, waiting time, and service time in ED

Table 1 shows that the mean overall length of stay (LOS) for the observed 100 patients was 194.09 minutes (SE = 19.21). Additionally, the mean waiting time was 157.87 minutes (SE = 14.38), constituting 77.7% of the total LOS in the ED. Conversely, the mean service time was 36.22 minutes (SE = 4.83), accounting for 22.3% of the overall LOS.

**Table 1:** Overall mean in minutes and score percentage of wait time, service time, and LOS estimated between the ED processes of the observed patients (*N*= 100)

**Item**	**Mean in minutes (SE)**	**Mean Score Percentage**
Overall wait time	157.87±14.38	77.7%
Overall service time	36.22±4.83	22.3%
Overall length of stay (LOS)	194.09±19.21	100%

### Analysis of waiting time across ed process phases

Table 2 and figure 2 reveal that the total waiting time across the five main time intervals was 8,397.37 minutes, with a mean waiting time of 83.97 minutes (SD = 75.49). The longest waiting time occurred during the consultation phase, totaling 7,027.05 minutes, with a mean waiting time of 92.46 minutes (SD = 59.28). This was followed by the decision disposition phase, with a delay of 920.34 minutes and a mean time of 9.20 minutes (SD = 16.40) between intervention and disposition decision. In contrast, the registration phase had the shortest delay, totaling 66.69 minutes, with a mean waiting time of 0.67 minutes (SD = 0.13).

**Table 2:** Total time in minutes, mean in minutes, and mean score percentage of waiting time within the ED processes (*N*=100 patients)

**ED Processes/patients**	**Total wait time in minutes**	**Mean in minutes (SE)**	**Mean % percentage**
Registration	66.96	0.67 (0.13)	0.80%
Initial screening	114.12	1.14 (0.25)	1.36%
Consultation	7027.05	92.46 (6.80)	83.68%
Interventions	268.9	3.02 (0.56)	3.20%
Disposition decision	920.34	9.20 (1.64)	10.96%
**Overall Wait Time**	**8397.37**	**83.97 (7.55)**	**100%**

### Patient satisfaction with waiting times across ED processes

Table 3 shows that the overall mean satisfaction score for patients was 61.74%, indicating a moderate level of satisfaction with the ED waiting times. The highest mean satisfaction scores were reported for the time taken before the first interaction with the assigned nurse and doctor, the time taken before being examined by a doctor, registration with the receptionist, and the total time spent in the ED. Conversely, the lowest satisfaction scores were associated with the waiting time for the consultant and the time taken to complete administrative processes. See Table 3 for more values.

**Table 3:** Patients’ satisfaction level related to their ED visit (*n*= 90) (VOC)

Items	Mean ±.SD	Rank
1. Time was taken from registration with a receptionist	3.98±.93	4
2. Time was taken before the first speaking with the assigned nurse.	4.41±.49	1
3. Time was taken before first speaking with the assigned doctor.	4.41±.49	1
4. Time was taken before being examined by a nurse	4.34±.65	2
5. Time was taken before being examined by a doctor	4.18±.83	3
6. Time was taken from requesting laboratory investigations to receiving the report.	3.22±1.42	6
7. Time was taken from requesting a diagnostic procedure to receiving the report.	3.20±1.38	7
8. Time was taken from the drug prescription from the hospital to the administration.	3.10±1.40	8
9. Time was taken from requesting to receiving/checking by specialty consultants	2.95±1.40	9
10. Time was taken to complete the administrative process.	2.64±1.55	10
11.In general, the total time of your visit to the emergency department	3.72±1.04	5
** Mean score %**	61.74.%	

### Staff satisfaction with ED Services

Table 4 demonstrates the satisfaction levels of ED staff regarding available resources, with an overall mean satisfaction score of 71.09%, reflecting a generally moderate level of satisfaction. The highest satisfaction scores were reported for available supplies, the number of pharmacists, interpersonal relationships among staff, and the number of nurses. Conversely, the lowest satisfaction scores were associated with bed capacity, emergency department design, and patients’ waiting time, highlighting critical areas that require improvement. See Table 4 for more values.

**Table 4:** Staff satisfaction related to resources in ED (VOB) (*N*=64)

Item	Mean ±.SD	Rank
Beds capacity	2.90±0.83	12
Available equipment & facilities	3.56±0.84	6
Available supplies	4.21±0.55	1
Number of doctors	3.34±0.90	8
Number of nurses	3.93±.67	4
Number of pharmacists	4.21±0.61	2
Patients’ waiting time	3.03±0.93	10
Patients’ paperwork	3.12±0.94	9
Interpersonal relationships among staff	4.00±0.76	3
Communication between staff and patients	3.81±0.78	5
Emergency department design	3.03±0.99	11
The environment of the emergency room is hygienic	3.46±0.76	7
**Mean score %**	71.09 %	

*SD* Standard deviation

### Staff-reported causes of extended waiting times in the ED

Table 5 highlights key factors contributing to extended waiting times in the emergency department, as reported by ED staff.

In terms of people-related causes, the most commonly cited issue was the lack of adherence to a triage protocol, reported by 95.0% of the staff. Other significant factors included an increase in non-urgent visits to the ED (83.3%), lack of awareness among patients and their relatives regarding the ED environment (66.7%), and a shortage of skilled triage nurses (43.3%). Regarding management-related causes, 50.0% of the staff identified the absence of scheduled training programs on patient triage as a factor contributing to longer waiting times. Process-related causes were also significant contributors to extended waiting times. The lack of an integrated care pathway for patients in the ED was highlighted by 90.3% of staff, along with non-adherence to the ED admission policy by patients and their families, leading to overcrowding (90.3%).

**Table 5:** Frequency distribution of the root causes of increased waiting time from VOB perspectives (*N*=64)

**Causes**	**N (%)**
**1: Causes related to people (*n*= 60)**	
No adherence to a triage protocol	57 (95)
Increase non-urgent visits	53 (83.3)
Lack of patients' and relatives' awareness about the ED environment	40 (66.7)
Lack of skilled triage nurses	26 (43.3)
Increase turnover nurses	16 (26.7)
Shortage of ED physicians	10 (16.7)
Contradictory opinion between ED staff and radiologist	8 (13.3)
The reluctance of some medical staff to carry out their responsibilities	4 (6.7)
**2: Causes related to management (*n*= 52)**	
No scheduled training programs related to patient triage.	26 (50.0)
No effective reward and punishment system.	18 (34.6)
Deputies take a long time to communicate with CT consultants.	7 (13.5)
There is no effective administrative system within the ED.	3 (5.7)
No policy for insured patients for faster response.	3 (5.7)
Insured patients’ reimbursement problems.	2 (3.8)
No defined policy for communication between ED staff and the X-ray department	1 (1.9)
**3: Causes related to process (*n*= 62)**	
No integrated care pathways for patients undergoing in the ED.	56 (90.3)
Non-adherence of the patients and their families to the ED admission policy leads to overcrowding.	56 (90.3)
Delay decision from the ICU consultant regarding whether to admit to the ICU	46 (74.19)
Delay in verifying the data of the privately insured patients.	44 (70.96)
Duplication of work between the system used for data entry and the accounting department	12 (19.3)
Poor communication between healthcare providers with patients.	4 (6.4)
No supervision by the infection control team periodically	2 (3.2)
No effective assignment to nurses on the patients.	2 (3.2)
Filling papers of patient records by the receptionist takes a lot of time.	2 (3.2)
Communication with other specialists takes a long time.	2 (3.2)
Poor communication between healthcare providers.	2 (3.2)
Delay to conduct radiology tests.	2 (3.2)
**4: Causes related to the environment (*n*= 64)**	
The presence of an excessive number of patients’ relatives	52 (81.3)
Not enough space for triage	52 (81.3)
Violation against staff from patients' relatives due to insufficient security members.	26 (40.6)
Ventilation problems (central air conditioner)	24 (37.5)
Bad phone network	20 (31.3)
Noise	16 (25.0)
No place for rest for staff	10 (15.6)
Not enough distance between beds	8 (12.5)
No nursing station in the unit	6 (9.4)
The presence of only one area for medication preparation	2 (3.1)
**5: Causes related to facilities (*N*= 42)**	
Insufficient number of beds	39(92.85)
Lack of maintenance of the machines	35(83.33)
Inadequate equipment and supplies.	20 (47.6)
Overload on the CT machine	10 (23.8)
The system of data entry is sometimes not working	8 (19.0)
Insufficient number of beds	4 (9.5)

Additionally, delays in decision-making by ICU consultants regarding admissions (74.19%) and delays in verifying the data of privately insured patients (70.96%) were reported as process inefficiencies that impact patient flow. Environment-related causes were identified as significant factors affecting patient wait times. Staff reported that an excessive number of patients’ relatives (81.0%) and inadequate space for triage (81.3%) contributed to delays. Additionally, 41.0% of staff reported incidents of violence from patients' relatives due to insufficient security personnel, which disrupts the ED environment. In terms of facilities-related causes, staff reported that an insufficient number of beds (92.85%) was a primary contributor to extended waiting times. Other issues included the lack of maintenance for machines (83.33%) and inadequate equipment and supplies (47.60%).

### Recommendations for improving ED processes and reducing waiting times

Table 6 highlights the key recommendations provided by ED staff to improve patient flow and reduce waiting times in the department. The majority of staff (95.3%) emphasized the need to develop integrated care pathways for patients in the ED. Additionally, 90.6% advocated for an expanded and well-designed physical area dedicated to triage, while 85.93% suggested assigning the responsibility for requesting CT scans to front-line physicians to minimize delays associated with waiting for consultants. Furthermore, 62.5% recommended establishing a clear policy for efficient communication with insurance companies to streamline administrative processes.

**Table 6:** Suggested improvement for the current patient flow (*N*=64)

**Items**	**N (%)**
Develop integrated care pathways for patients undergoing in the ED.	61 (95.3)
Extending a well-designed physical area of triaging	58 (90.6)
Assigning the responsibility of requesting the CT scan to the front-line physicians to avoid long waiting times for consultants	55 (85.93)
Developing a policy for smart communication with insured companies.	40 (62.5)
Developing a robust data capture system by working with the IT department is that data can be more easily extracted for analysis.	34 (53.1)
Increasing the number of beds in the unit.	30 (46.9)
Provision of scheduled training programs for doctors and nurses about emergencies such as (triage, ALS, ATLS, and ACLS).	28 (43.8)
Developing a policy for controlling crowdedness due to accompanying patients’ family members in the ED and increasing security personnel.	24 (37.5)
Provide periodic maintenance of machines and equipment.	18 (28.1)
Policy for standardizing communication between ED staff and other specialties.	16 (25.0)
Consider increasing the number of ambulances to speed up patient transfer.	14 (21.9)
Emphasizing and disseminating the guidelines to people about the emergency department and communication with caregivers.	6 (9.4)
Provide a nursing station in the unit.	4 (6.3)
Policy for communication between the ER team and other specialties.	4 (6.3)
Hire qualified triage nurses.	2 (3.1)
Hire more specialty consultants such as urology and neuropsychology.	2 (3.1)

Beyond these primary recommendations, 53.1% of staff supported using tele-technology (telemedicine) to facilitate faster access to radiology consultants, enhancing the efficiency of diagnostic services. Another 46.9% emphasized the importance of increasing the number of beds in the unit to accommodate patient demand, while 43.8% suggested implementing scheduled training programs for nurses and doctors on emergency topics, including triage, Advanced Life Support (ALS), Advanced Trauma Life Support (ATLS), and Advanced Cardiac Life Support (ACLS). Additionally, 37.5% of the staff expressed the need for increased security personnel to maintain order and safety within the ED. Meanwhile, 28.1% recommended regular maintenance of machines and equipment and increasing the number of healthcare providers to improve service capacity. Finally, one-quarter (25.0%) of the staff advocated for the development of a standardized communication policy between ED staff and other specialties to ensure effective collaboration.

## Discussion

This study evaluated patient flow in an emergency department (ED) using a Lean-integrated approach, examining key performance indicators (KPIs) such as wait time, service time, and length of stay to capture the Voice of Process, as well as the Voice of Customer (patient satisfaction) and Voice of Business (staff perspectives). The findings provided a comprehensive assessment of patient flow challenges, revealing critical areas for improvement, particularly extended waiting times across various ED phases, which significantly impacted both patient experience and operational efficiency. An A3 report was also developed to propose targeted strategies for reducing waiting times and improving overall patient flow, aiming to enhance the experience for both patients and staff in the ED.

### Voice of Process (VOP)

#### Patient journey and process flow in the emergency department

*The present study revealed that the patient journey in the ED consists of five structured phases:* admission and registration, initial assessment, consultation, intervention, and decision disposition. This structured process aligns with Lean methodology's emphasis on minimizing variability and enhancing operational efficiency [13]. The standardization observed in the studied hospital may be attributed to the presence of a well-defined ED policy supported by detailed procedures for each phase. Additionally, the triage process was governed by a structured triage policy based on the Australasian Triage Scale (ATS). Supporting this approach, Ortíz-Barrios and Alfaro-Saíz [12] emphasized the importance of structured pathways for improving ED flow, as they facilitate the identification of bottlenecks and allow for targeted process improvements to mitigate delays. Similarly, Heufel et al. [41] noted that clear, standardized care pathways in the ED can reduce bottlenecks and streamline processes, thereby enhancing patient flow and operational efficiency.

#### Length of stay, waiting time, and service time

*The findings of this study revealed that the overall length of stay (LOS) for the 100 patients observed in the ED exceeded 3 hours, with over 2.5 hours (77.7% of the total LOS) spent in non-value-added waiting time or “waste.”* This significant proportion of waiting time aligns with Lean principles, which emphasize identifying and eliminating inefficiencies to streamline operations. This delay could be likely stem from inefficiencies in critical ED processes, including prolonged wait times for radiology examinations, consultations, decision disposition, and health insurance payment approvals. Such inefficiencies not only negatively affect patient satisfaction but also increase the workload on ED staff, as highlighted in prior studies by Mostafa and El-Atawi [42]. Similar findings were reported by Simkhada et al. [43], who observed an average ED LOS of 3.18 hours. In contrast, Singh et al. documented a median ED LOS of 1.75 hours, potentially reflecting variations in ED efficiency and resource allocation. Furthermore, Paling et al. [33] noted that up to 31% of ED patients experienced LOS exceeding 4 hours, underscoring the variability across healthcare settings and emphasizing the need for operational benchmarking and tailored interventions.

*Moreover, the findings revealed that the consultation phase accounted for the most significant delays, with a cumulative waiting time of 7,027.05 minutes across all patients,* highlighting it as a critical area for improvement. Prolonged consultation durations were primarily attributed to waiting for test results, imaging reports, or specialist opinions, and inconclusive results that often necessitated additional diagnostic procedures, further compounding delays. This result aligns with the findings of van der Veen et al. [44], who identified consultation delays as a major bottleneck caused by limited specialist availability and prolonged diagnostic processing times. From the authors' perspective, these delays underscore the need for structural and procedural enhancements within the ED. Therefore, implementing telemedicine consultations, as recommended by Arnaud et al. [45], offers a viable solution to expedite access to specialists, streamline diagnostic workflows, and significantly reduce consultation times. Focusing on targeted improvements in this phase could yield substantial reductions in overall ED wait times, ultimately enhancing patient flow, satisfaction, and operational efficiency.

Furthermore, the present study revealed that longer ED service times and extended lengths of stay for level II triage patients may be attributed to these patients often presenting with unclear clinical symptoms that neither clearly indicate the need for admission nor discharge. As a result, these patients require additional time for thorough evaluation, investigation, and treatment in the ED. These findings align with those of Boudi et al. [46], who reported that critically ill patients (levels I and II) frequently experience quick initial evaluations but prolonged lengths of stay, consistent with Canadian Triage Assessment Scale (CTAS) objectives [47]. This extended care time is anticipated, as managing urgent conditions often necessitates comprehensive care [48]. Similarly, Kang et al. [49] observed an increase in higher acuity cases (CTAS 1 and 2) alongside a decrease in lower acuity presentations (CTAS 4 and 5), with 83% of high-acuity patients facing significant waiting delays. These findings highlight the critical need for effective triage protocols and prioritization strategies to optimize patient flow in EDs handling high-acuity patient populations.

Further supporting these observations, van der Veen et al. [44] reported that ED stagnation often arises from patients requiring input from multiple specialties or radiological imaging. Similarly, other studies identified laboratory and radiology services, consultation delays, and bed shortages as significant sources of ED inefficiencies [50, 51]. This study’s findings align with these observations, showing the longest delays were linked to diagnostic services and extended LOS.

Such delays may result from inadequate coordination between the ED and other hospital departments. Assigning radiological responsibilities to frontline doctors rather than consultants, as indicated in this study, could reduce wait times [48]. Additionally, Lai et al. [52] found that 10.1% of patients experienced consultation delays, highlighting their impact on ED efficiency. These findings underscore the need for improved coordination and streamlined processes to enhance ED efficiency.

### Voice of Customer (VOC)

#### Patient satisfaction with waiting times

Although the waiting times recorded in this study appear shorter than those reported in some previous studies, the VOC assessment indicated moderate patient satisfaction with ED waiting times. While patients expressed higher satisfaction with initial interactions, their satisfaction declined significantly during extended wait times for consultations and administrative procedures, negatively affecting their overall ED experience. This may be attributed to the prioritization of life-threatening cases over less critical conditions, leaving non-urgent patients to endure longer waits. These findings align with Xie and Or [28], who reported that extended wait times for specialist consultations adversely impact patient satisfaction. Additionally, Mostafa and El-Atawi [42] emphasized the importance of timely initial assessments and effective communication about expected wait times to maintain patient satisfaction. Consistent with these insights, this study highlights the critical need for managing patient expectations and improving communication regarding wait times.

### Voice of Business (VOB)

#### Staff satisfaction with ED services

The current study revealed moderate staff satisfaction levels with ED resources. Staff expressed strong satisfaction with supplies, staffing, and interpersonal relationships but reported lower satisfaction with bed capacity and patient waiting times. These findings may stem from treatment delays caused by bed shortages, which not only frustrate patients—often leading to complaints or conflicts with healthcare providers—but also pressure staff to expedite procedures, potentially compromising the quality of care. From the authors' perspective, addressing these challenges is crucial for enhancing ED operations and staff well-being. The results align with Heufel et al. [41], who emphasized resource limitations and patient flow inefficiencies as significant concerns for ED staff. Bijani and Khaleghi [53] also highlighted the importance of increasing bed capacity and optimizing staffing to reduce workloads and improve operational balance. Therefore, targeted interventions, such as expanding bed availability and enhancing resource allocation, could alleviate these challenges, improving patient throughput and overall ED efficiency.

#### Staff-reported causes of extended waiting times in the ED

Healthcare providers in this study identified multiple factors contributing to extended waiting times in the ED, including non-adherence to triage protocols, increased non-urgent visits, and insufficient triage space. Additionally, management-related issues, such as a lack of scheduled training programs and delays in ICU consultant decisions, were significant contributors. Overcrowding, exacerbated by an excessive presence of patients' relatives and limited triage space, further hindered care delivery. Limited patient and family awareness of ED procedures, a shortage of skilled triage nurses, and overcrowding due to non-compliance with admission policies compounded these delays. Physical constraints, such as inadequate bed capacity, equipment maintenance issues, and insufficient supplies, were also highlighted as barriers to effective ED operations.

From the authors' perspective, these findings underscore a critical need to strengthen triage protocols and patient education programs to address non-compliance and reduce the burden of non-urgent visits. The absence of adequate triage spaces and specialized staff training likely reflects systemic gaps in ED infrastructure and resource allocation. Addressing these issues could lead to significant improvements in patient flow and care delivery.

These findings align with Bijani and Khaleghi [53], who highlighted limited physical space, insufficient triage nurses, and inadequate security resources as major challenges in ED environments. Heufel et al. [41] emphasized that the absence of standardized screening and care delivery protocols results in inconsistent interventions, hindering the evaluation of ED performance. Similarly, Al-Otmy et al. [47] reported that common reasons for non-urgent ED visits include perceived urgency (42.0%), ease of access (25.5%), and limited services at public healthcare centers (17.8%), emphasizing the need for enhanced primary care access and public awareness to reduce unnecessary ED use.

The authors believe that addressing structural and procedural inefficiencies could yield significant benefits. For instance, integrating targeted education programs for patients and families could reduce non-urgent ED visits, allowing staff to focus on critical cases. Additionally, improving interdepartmental communication and assigning radiological and diagnostic responsibilities to frontline doctors could mitigate delays in consultations and diagnostic services. These challenges resonate with previous research showing that EDs often accommodate patients with varying levels of urgency, resulting in delays for those in lower triage categories [17]. Logistical barriers, such as insufficient resources and time constraints, further complicate care quality [15–18].

#### Recommendations for improving ED processes

Based on staff feedback and voice, the study identified several key recommendations for enhancing ED processes and reducing waiting times. These suggestions were grounded in practical insights from those directly involved in ED operations, ensuring that the proposed solutions address real-world challenges. Recommendations included developing integrated care pathways, expanding triage spaces, enabling front-line physicians to directly request CT scans, and establishing efficient communication protocols with insurance companies. Incorporating Lean principles offers a structured approach to reduce waste, optimize resources, and enhance satisfaction for patients and staff, fostering a more efficient, patient-centered environment [54].

These recommendations align with findings from van der Velde et al. [55], who emphasized that structured care pathways can improve ED efficiency and care quality. Similarly, Bijani and Khaleghi [53] advocate for empowering triage nurses and physicians and addressing structural issues in triage units, as these changes can improve the effectiveness and quality of triage processes. Moreover, Kang et al. [49] highlighted the importance of efficient admission processes, which can help reduce waiting times for both admitted and discharged ED patients. Their study supports the use of telemedicine to enhance ED operations, suggesting that clear admission protocols and frameworks are essential for effective policy implementation. Li et al. [52] further emphasized the need to minimize patient waiting times, noting that patients often wait longer than they receive actual care. In their research, telemedicine successfully shortened wait times by offering remote expertise, thereby improving the timeliness of care delivery in settings with limited on-site resources.

### Strengths and limitations

This study provides valuable insights into the causes of delays in an emergency department (ED) and offers actionable recommendations to improve patient flow and satisfaction. A major strength of the study is its comprehensive approach, integrating both quantitative and qualitative data from patients and ED staff. This dual perspective enables a detailed understanding of patient flow challenges and the identification of practical interventions to address inefficiencies. The use of a mixed-methods design and real-time observations of patient pathways enhances the validity of the findings by directly capturing ED processes and staff perspectives.

However, several contextual factors and methodological limitations may have influenced the results. First, the study was conducted in a single private hospital, where resource availability and patient demographics may differ significantly from public hospitals, potentially limiting the generalizability of the findings. Private hospitals often have more robust staffing and resource allocations, which might mitigate some challenges faced by public facilities [54]. Second, the focus on specific time intervals, from registration to disposition decision, excluded data on total length of stay (LOS) for patients who were eventually discharged or transferred. This may have overlooked key factors influencing total LOS, such as post-decision delays or patient-specific clinical needs. Third, the lack of assessment of clinical care quality outcomes limits the study’s ability to evaluate the broader impact of delays on patient safety and treatment effectiveness.

These contextual and methodological factors highlight the need for cautious interpretation of the findings. Future research could address these limitations by including diverse hospital settings, capturing comprehensive LOS data, and incorporating clinical care outcomes to provide a more nuanced understanding of ED performance.

## Conclusion

This study highlights significant factors contributing to delays in emergency department (ED) operations, including non-urgent visits, gaps in triage protocols, space limitations, staffing shortages, and inefficiencies in coordination and procedural workflows. These delays disproportionately extend patient waiting times, particularly in the consultation phase, which emerged as the most critical bottleneck. From the findings, patient satisfaction was generally high for initial interactions but decreased significantly for extended wait times during consultations and administrative processes. Similarly, staff expressed satisfaction with resources and interpersonal dynamics but pointed to bed capacity, patient wait times, and documentation processes as key areas needing improvement.

The results underscore the need for targeted strategies such as implementing integrated care pathways, optimizing triage systems, leveraging telemedicine for specialist consultations, and enhancing interdepartmental communication to address systemic inefficiencies. Furthermore, improving staff training, expanding resources, and redesigning ED layouts can mitigate structural constraints, reduce delays, and enhance patient flow. Addressing these structural, procedural, and technological gaps requires collaboration among ED administrators, hospital management, and healthcare providers. By addressing these challenges, EDs can significantly improve patient and staff satisfaction while fostering a more efficient and responsive healthcare environment.

### Implications of the study

#### Implications for practices and policies

This study highlights actionable implications for improving Emergency Department (ED) operations at both practice and policy levels.

From a practice perspective, Emergency Department managers and clinical leaders should focus on implementing integrated care pathways and streamlined patient flow processes to reduce delays and improve coordination. Expanding triage capacity and empowering front-line physicians to independently request diagnostic imaging, such as CT scans, can minimize bottlenecks caused by consultant availability. Leveraging telemedicine for faster specialist consultations and enhancing staff training on emergency protocols like ALS, ATLS, and ACLS should be spearheaded by training coordinators and supported by hospital management.

At the policy level, hospital administrators and healthcare policymakers should address staffing shortages by creating optimized hiring policies for healthcare professionals and security personnel. They should also focus on standardizing interdepartmental communication protocols and streamlining interactions with external stakeholders, such as insurance companies, to reduce administrative delays. Ensuring regular maintenance of medical equipment and increasing bed capacity requires collaborative efforts from hospital maintenance teams and healthcare administrators, ensuring resources are allocated effectively.

By integrating these practice-focused and policy-driven interventions, healthcare organizations, Emergency Department managers, and policymakers can work together to enhance efficiency, reduce waiting times, and create a more patient-centered and supportive care environment.

#### Recommendations for future research

Future research should evaluate integrated care pathways and streamlined triage systems across diverse healthcare settings to determine their scalability and effectiveness. Comparative studies on different staffing models and resource allocations could provide insights into optimizing ED performance. Additionally, exploring telemedicine's role in expediting diagnostics and care delivery in resource-limited EDs is essential.

Research on ED design and its impact on patient flow and staff efficiency, along with the long-term effects of maintenance, equipment upgrades, and staff training, could identify sustainable solutions. Lastly, assessing the impact of patient and family education programs on reducing non-urgent ED visits may guide policies for better resource utilization.

## Supplementary Information

Supplementary Material 1

## Data Availability

Data is provided within the manuscript or supplementary information files.

## Abbreviations

ACLS: Advanced Cardiac Life Support
ALS: Advanced Life Support
ANOVA: Analysis of Variance
ATLS: Advanced Trauma Life Support
CT: Computed Tomography
CTAS: Canadian Triage Assessment Scale
ED: Emergency Department
ICU: Intensive Care Unit
IRB: Institutional Review Board
JC: Joint Commission
LOS: Length of Stay
NHS: National Health Service
PHC: Public Healthcare Center
SD: Standard Deviation
SPSS: Statistical Package for the Social Sciences
VOB: Voice of Business
VOC: Voice of Customer
VOP: Voice of Process
