# Supplementary material for: Streamlining emergency nursing care post-pandemic: A lean approach for reducing wait times and improving patient and staff satisfaction in the hospital
Source: BMC Nurs. 2025 Apr 22;24:445. doi: 10.1186/s12912-025-02759-w (PMC12016415; doi:10.1186/s12912-025-02759-w)
Supplement: Supplementary file 1 — Supplementary Material 1 [file 12912_2025_2759_MOESM1_ESM.zip › A3 tool.pdf]

Title: **Sponsor:** ……………..

Author: Researchers **Date:** ………

1. The theme/title: ……………………………………………………………………

2. Background:

3. Current Condition:

4. Goal:

5. Root Causes Analysis:

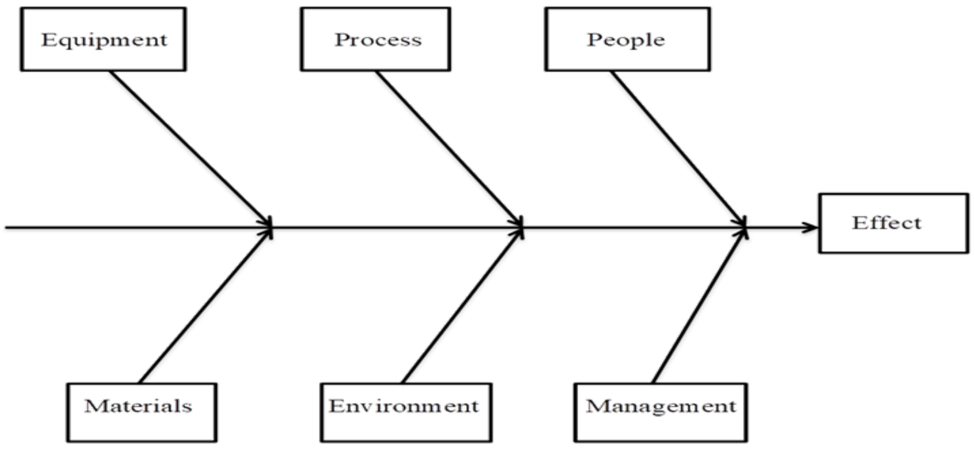

Item
**1: Causes related to people (n= 30)**<br>Lack of skilled doctors<br>Lack of skilled triage nurses<br>Increase turnover nurses<br>Lack of patients and relatives' awareness about ED environment<br>The reluctance of some medical staff to carry out their responsibilities<br>Increase non-urgent visits<br>Contradictory opinion between ED staff and radiologist<br>Shortage of ED physicians<br>No adherence to triage protocol<br>Insured company employee reluctance or not present in his office
**2: Causes related to management (n= 52)**<br>No effective reward and punishment system<br>No effective training programs related to emergency as triage<br>There is no administrative system within the ED<br>No defined policy for communication between ED staff and CT consultant<br>Deputies take long time to communicate with CT consultant<br>No policy for insured patients for faster response<br>Accounting problems
**3: Causes related to process (n=**<br>No defined pathway for patients in ED<br>COVID protocol leads to increased waiting time<br>Private insurance problems (delay response from most of insured companies)<br>Delay decision from ICU consultant regarding whether to admit to ICU<br>Duplication of work between system used for data entry and accounting department<br>No supervision by infection control team periodically<br>No effective assignment to nurses on the patients<br>Filling papers of patient record by the receptionist take a lot of time<br>Communication with other specialists take long time<br>Triage is activated for measuring vital signs only not for sorting patients<br>Time taken to persuade patients and relatives about COVID protocol<br>Poor communication between healthcare providers<br>Poor communication of healthcare providers with patients<br>Delay to conduct radiology tests
**4: Causes related to Environment**<br>No place for rest for staff<br>No enough distance between beds<br>Presence of only one area for medication preparation<br>Presence of an excessive number of patients' relatives<br>Ventilation problems (central air conditioner)<br>No security persons in the ED<br>Noise<br>Bad phone network<br>No nursing station in the unit<br>No dedicated section for triage
**5: Causes related to Equipment**<br>Some machines do not work effectively as sphygmomanometers<br>Lack of numbers of equipment needed<br>Overload on CT machine<br>The system of data entry is sometimes not working<br>Insufficient numbers of beds

6. Plan improvement goals

7. Implementation/Time line of plan implementation:

8. Follow up:

The researcher will follow up plan implementation.

Figure 21: A3 proposal report
