# Supplementary material for: Streamlining emergency nursing care post-pandemic: A lean approach for reducing wait times and improving patient and staff satisfaction in the hospital
Source: BMC Nurs. 2025 Apr 22;24:445. doi: 10.1186/s12912-025-02759-w (PMC12016415; doi:10.1186/s12912-025-02759-w)
Supplement: Supplementary file 1 — Supplementary Material 1 [file 12912_2025_2759_MOESM1_ESM.zip › Lean 1-2Tools.pdf]

Tool (I) Voice of Customer (VOC) Structured Interview

A. Socio-demographic and clinical data

1. Name: ………………………………………………………….….
2. Age: …………………………………………………….………
3. Sex: male ( ) female ( )
4. Occupation: ……………………………………….……………
5. Educational level:

   - Illiterate ( ) - Lower education ( ) Average education ( ) - Bachelor ( )

   - Diploma - Master ( ) - Doctorate ( ).
6. Residence: ……………………….……………………………
7. Do you have medical history: No ( ) Yes ( )

If yes

- What is it? …………………………………………………….
- Since when? …………………………………………………..

8. Do you have surgical history: No ( ) Yes ( ).

   If yes

- What is it? …………………………………………………...
- Since when? ………………………………………………….

9. Your complain: …………………………..…………………..
10. Do you have health insurance? No ( ) Yes ( )

B. **Patients' waiting time in Emergency Department**

1. **Were you informed about expected waiting time you will spend in Emergency Department?**

   a. No

   b. Yes

   If yes:

   b. 1. The time was shorter than expected

   b. 2. The time was as expected

   b. 3. The time was longer than expected

2. **Please determine how long you did wait in the following stages and degree of satisfaction regarding them:**

	**Stages**	**Strongly Dissatisfied (1)**	**Dissatisfied (2)**	**Neutral (3)**	**Satisfied (4)**	**Strongly Satisfied (5)**
1	Registration with system employee<br>Waiting time: ……………					
2	Before you first spoke to a nurse<br>Waiting time: ……………					
3	Before you first spoke to a doctor<br>Waiting time: ……………					
4	Before being examined by a nurse<br>Waiting time: ……………					
5	Before being examined by a doctor<br>Waiting time: ……………					
6	For laboratory services from the request to the result arrival<br>Waiting time: ……………					
7	For drugs from hospital from the prescription to administration<br>Waiting time: ……………					
8	For your company to approve your admission to hospital unit as a health insurance case<br>Waiting time: ……………					
9	For consultation from doctor order to examination<br>Waiting time: ……………					

10	In general, about how long it took you to visit the emergency department.<br>Waiting time: ……………					

3. Did you have enough time to discuss your health problem with the nurse?

a. Yes, definitely

b. Yes, to some extent

c. No

If no, why?

....

....

....

....

....

4. Did you have enough time to discuss your health problem with the doctor?

a. Yes, definitely

b. Yes, to some extent

c. No

If no, why?

....

....

....

....

....

Tool II: Voice of Business (VOB) Semi-Structured Interview:

Please answer these questions:

A. Personal data:

1. Current employment position: ………………………….
2. Age: ……………………………………….……………….
3. Years of experience since graduation: …………………………
4. Years of experience in current organization: ………………......

B. Emergency Department staff's perceptions to improve patient flow and decrease waiting time:

1. Please determine degree of your satisfaction regarding:

	Items	Strongly Dissatisfied (1)	Dissatisfied (2)	Neutral (3)	Satisfied (4)	Strongly Satisfied (5)
I	Beds capacity in the Emergency Department in relation to admission rate.					
II	Available equipment for required procedures in the Emergency Department.					
III	Available supplies for required procedures in the Emergency Department.					
IX	Numbers of doctors in the Emergency Department.					
X	Numbers of nurses in the Emergency Department.					
XI	Numbers of pharmacist in the Emergency Department.					
XII	Patients' waiting time in the Emergency Department.					
XIII	Patients' paperwork in the Emergency Department.					
XIV	Interpersonal relationship between staff in Emergency Department.					

XV	Communication between staff and patients.					
XVI	Emergency Department Design.					
XVII	Emergency Department as whole.					

2. Is there any budget for Emergency Department renewal/ improving?

I. Yes
II. No
III. Do not know

3. Is there a job description?

I. Yes
II. No

4. Did you attend lean training program before?

I. Yes
II. No

5. Are you need for training program in quality, lean or Emergency?

I. Yes
II. No

6. From your perspective what are the causes of increased waiting time in Emergency Department, you can choose more than one option?

I. Overcrowding by the family of patients
II. Shortage of Emergency Department's nurses
III. Lack of trained triage nurses
IV. Shortage of Emergency Department Physicians
V. Insufficient resources
VI. Others

....

....

....

....

....

7. Is there a policy regarding waiting time for the patient at each stage of treatment in the emergency department?

I. Yes

II. No

III. Do not know.

If yes

- Were you notified of it upon appointment? Yes ( ) No ( ).
- Has there been any change in it? Yes ( ) No ( ).

8. From your perspective, how this problem affects hospital's goals or values?

....

....

....

....

....

9. From your perspective, what are the root causes of increased waiting time?

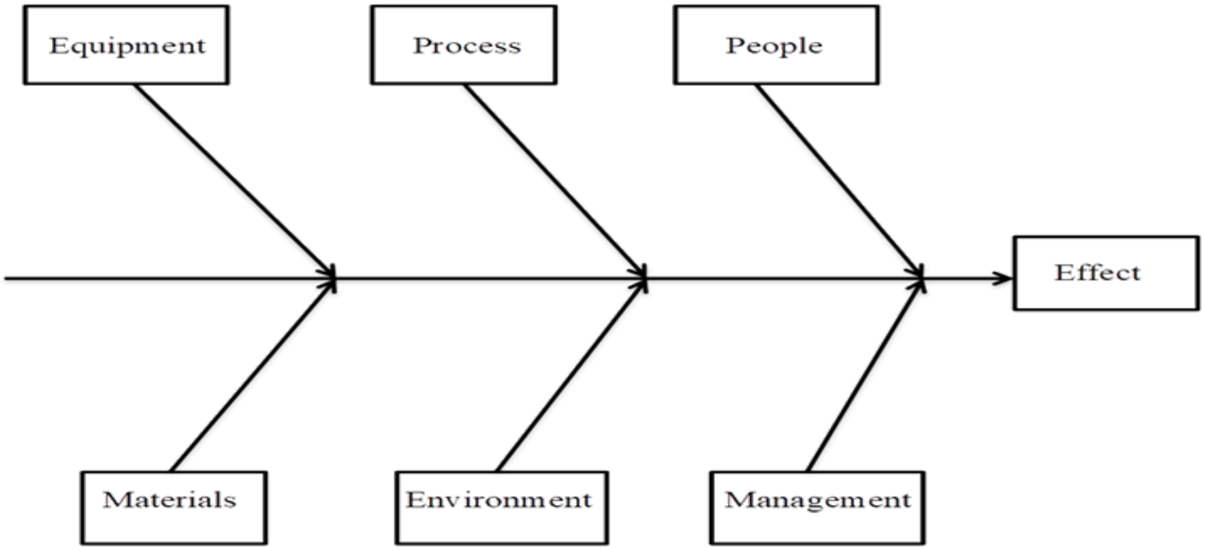

10. From your perspective, how to improve the current patient flow?

....

....
