# Supplementary material for: Streamlining emergency nursing care post-pandemic: A lean approach for reducing wait times and improving patient and staff satisfaction in the hospital
Source: BMC Nurs. 2025 Apr 22;24:445. doi: 10.1186/s12912-025-02759-w (PMC12016415; doi:10.1186/s12912-025-02759-w)
Supplement: Supplementary file 1 — Supplementary Material 1 [file 12912_2025_2759_MOESM1_ESM.zip › Multi voting recomendation sheet.pdf]

Multi-voting of the collected recommendation :

Please read the following recommendation items carfully and place adot by the option you belife should be worth for improving the emergency departemnt waiting time a

Items	Vote count	Number of vote
Developing policy for communicating with insured companies		
Developing a robust data capture system by working with the IT department is that data can be more easily extracted for analysis.		
Increasing numbers of beds in the unit		
Provision/ providing training program for doctors and nursing about emergency as (triage, ALS, ATLS and ACLS)		
Developing policy for controlling crowdedness due to accompanied patient’s family members in the ED and increase security personnel.		
Provide periodic maintenance of machines and equipment		
Policy for standardizing communication between ED staff and other specialties		
Consider increasing number of ambulances to speed patient transfer		
Develop integrated care pathway for patients undergoing in the ED		
Making guidelines to educate people about the emergency department and communication with caregivers		
Provide nursing station in the unit		
Policy for communication between ER team and other specialties		
Hire qualified triage nurses		
Provide section for triage		
Hire more specialty consultant as urology and neuropsychology		
