# Supplementary material for: Streamlining emergency nursing care post-pandemic: A lean approach for reducing wait times and improving patient and staff satisfaction in the hospital
Source: BMC Nurs. 2025 Apr 22;24:445. doi: 10.1186/s12912-025-02759-w (PMC12016415; doi:10.1186/s12912-025-02759-w)
Supplement: Supplementary file 1 — Supplementary Material 1 [file 12912_2025_2759_MOESM1_ESM.zip › Supplementary Materials lean.docx]

**Supplementary**

**Supplementary-Table I.** *Frequency distribution of the clinical data of the observed patients passed through ED processes (N= 100)*

| **Items** | **N (100)** | **(%)** |
| --- | --- | --- |
| **Modes of arrival** |  |  |
| Walk in  Ambulance  Wheelchair/private vehicles | 89  8  3 | 89%  8%  3% |
| **Triage category** |  |  |
| Category (1): Immediately life-threatening  Category (2): Imminently life-threatening  Category (3): Potentially life-threatening  Category (4): Potentially serious  Category (5): Less urgent | 2  48  25  12  13 | 2%  48%  25%  12%  13% |
| **Complaints/body system(s)** |  |  |
| Neurology  Respiratory  Cardiology  Accidents (e.g., RTA, and electric burn)  Others (e.g., Sepsis, and suspected appendicitis) | 32  8  32  7  21 | 32%  8%  32%  7%  21% |

**Supplementary Table 2:** *Frequency distribution of the interviewed patients according to their demographic, professional data, and clinical baseline characteristics (VOC-N=90).*

| **Items** | **n (90)** | **(%)** |
| --- | --- | --- |
| **Age**  <20 Years  20-  30-  40-  50 years and more | 6  6  6  24  48 | 6.66%  6.66%  6.66%  26.66%  53.33% |
| Mean (SD): 52.69 (17.73) | | |
| **Sex** |  |  |
| Male  Female | 62  28 | 68.0%  31.3% |
| **Residence** |  |  |
| Urban  Rural | 78  12 | 86.66%  13.33% |
| **Educational level** |  |  |
| Illiterate  Primary education  Secondary education  Associate degree  University and higher education (bachelor’s and/or  master’s degree) | 9  15  28  18  20 | 1.0%  16.66%  31.11%  20.0%  22.22 |
| **Occupation** |  |  |
| Housewife  Pension  Professional (Public employees)  Free work  Students | 25  25  19  19  2 | 27.7%  27.7%  21.11  21.11  2.22 |
| **Payer type**  Private insurance  Self-pay | 15  75 | 16.66%  83.33 |
